# 人附睾蛋白4在肺癌中的研究进展

**DOI:** 10.3779/j.issn.1009-3419.2015.03.10

**Published:** 2015-03-20

**Authors:** 晴 马, 倩 王, 殿胜 钟

**Affiliations:** 300052 天津，天津医科大学总医院肿瘤科 Department of Medical Oncology, The General Hospital of Tianjin Medical University, Tianjin 300052, China

**Keywords:** 人附睾蛋白4, 肺肿瘤, 进展, HE4, Lung neoplasms, Progress

## Abstract

人附睾蛋白4（human epididymis 4, HE4）属于乳清酸性4-二硫化中心（WFDC）蛋白家族，具有胰蛋白酶抑制剂的特性。在肺癌患者血清中及恶性胸腔积患者胸水中呈高表达，现有临床数据显示它与肺癌的诊断及预后有一定的相关性，因此可能成为临床上肺癌的诊断及预后评估的新指标。

## Abstract (en)

Human epididymis 4 (HE4) belongs to whey acidic 4-disulfide center protein family. It has the characteristics of inhibiting trypsin and high expression in the serum or malignant pleural effusion of patients with lung cancer. Clinical data showed that it has certain relevance to the diagnosis and prognosis of lung cancer. HE4 may be a new indicator of clinical diagnosis and prognosis evaluation.

肿瘤标记物是肿瘤发生、发展、浸润及转移过程中产生的一种特异性物质，可从肿瘤组织或宿主体液中检测到。在临床上，肿瘤标记物常常被用来进行肿瘤的诊断和随访。癌胚抗原（carcino-embryonic antigen, CEA）、细胞角蛋白-19（cytokerantin-19-fragment, CYFRA21-1）等标记物已被广泛应用于肺癌的诊断，但敏感性和特异性均不高。目前，有研究报道了人附睾蛋白4（human epididymis 4, HE4）在肺癌患者血清中及恶性胸腔积液患者胸水中的水平及其与诊断、预后的关系，为临床上肺癌的诊断及预后评估提供新的指标。

## 1 HE4的结构与表达

HE4属于乳清酸性4-二硫化中心（WFDC）蛋白家族，具有胰蛋白酶抑制剂的特性。此家族中的其他蛋白还包括SLPI、Elafin和PS20（WFDC1）^[1, 2]^。*HE4*基因位于人类基因组染色体20q12-13.1，编码一个13 kDa的蛋白，在其成熟的糖基化形成时，蛋白可达20 kDa-25 kDa，并且包括含有两个WFDC结构域的一条单链^[3]^。

HE4首先由Kirchhoff等^[4]^学者通过cDNA筛检在附睾组织中发现。Bingle等^[3]^学者通过Northern杂交方法在肺、肾及唾液腺中均发现了HE4 mRNA的表达。之后，有学者^[5]^证实，HE4在卵巢癌组织中过表达。在卵巢癌患者血清中也可以检测到高水平的分泌型HE4^[2]^。Galgano等^[6]^对175例肿瘤患者进行分析，发现*HE4*基因在卵巢浆液性癌中表达最高，在肺腺癌、乳腺癌、移行细胞癌及胰腺癌中中等甚至强阳性表达。另外，Ross等^[7]^学者也证实，HE4在一些肿瘤细胞系中也存在高表达，包括Ovcar-3和Ovcar-4（卵巢癌）、HT-29、HCT-116和COL0205（结肠癌）、MALME-3M（黑色素瘤）、MCF-7（乳腺癌）和A498以及786-0（肾癌）。这种肿瘤组织或细胞系中HE4的高表达，以妇科肿瘤和肺癌尤为明显。大量的研究也证实，血清HE4可以作为肿瘤标志物用于鉴别妇科良性肿瘤和卵巢癌，其结果与CA-125相似。因此，2008年美国FDA批准血清ELISA检测HE4作为辅助诊断卵巢癌的指标之一^[8-11]^。以上结果均提示，HE4可能是一种有价值的肿瘤标记物。

## 2 HE4在肺癌患者血清及组织中的表达及意义

在呼吸系统中，HE4在正常口腔上皮细胞、鼻咽，特别是气管中高表达，但在正常肺组织中的表达水平高低尚有争议。血清HE4表达水平受年龄以及吸烟状况影响，并与这两者呈正相关性。肾功能不全也可使血清HE4显著升高^[12]^。

肺癌患者血清HE4明显高于正常对照。Yamashita等^[13]^利用免疫测定技术分别对102例经病理确诊为原发性肺腺癌、16例肺部良性疾病及58例健康志愿者血清中HE4浓度进行检测，结果表明，肺癌患者血清中HE4水平明显较良性组及健康组升高，差异有统计学意义（*P*＜0.000, 1），血清HE4诊断肺癌的曲线下面积0.825，当血清HE4含量的临界值为50.3 pmmol/L时，诊断的敏感度特异度分别为74.5%和81%。

基于上述数据，有学者探讨了血清HE4对于肺部良、恶疾病鉴别的意义^[14]^，研究共纳入106名健康志愿者、109例肺癌患者、114例结核患者以及24例肺炎患者，肺癌患者血清HE4明显高于健康人和结核患者（*P*＜0.001）。当临界值为94.01 pmol/L时，从结核患者中鉴别肺癌的敏感性为61.6%，特异性为93.0%。调整年龄、吸烟影响，并进行二进制无条件逻辑回归后敏感性为67.4%，特异性为86.0%。而在肺炎患者中，由于炎症因子如IL-1可以诱导HE4生成，因此，HE4不能作为两者鉴别诊断的依据。该研究也提示肺癌患者中，与小细胞肺癌、鳞癌相比较，肺腺癌血清中HE4表达更高^[14, 15]^。这些数据都显示，HE4可能作为肺癌诊断和随访的新指标，并且在乳腺癌和卵巢癌中，血清HE4已经成为预测因子之一。

血清HE4与肺癌pTNM相关，在进展期肺癌患者呈高表达。Iwahori等^[16]^通过ELISA法对49例肺癌患者血清中HE4浓度进行测定，通过敏感性特异性分析发现，当临界值为6.56 ng/mL时，90%的非小细胞肺癌、88.9%的小细胞肺癌血清中HE4浓度升高；血清HE4诊断肺癌的受试者工作特征曲线（receiver operating characteristic curve, ROC）下面积达0.988。此外，进一步对其中24例化疗后肺癌患者生存率与HE4含量关系进行分析发现，部分缓解（patial response, PR）患者化疗前后HE4水平分别为23.0 ng/mL和12.6 ng/mL，疾病进展（progressive disease, PD）患者分别为21.3 ng/mL和23.8 ng/mL，化疗后PD患者较PR患者血清HE4水平增高（*P*＜0.001）。此外，以15 ng/mL为界，血清中低水平HE4患者的中位生存期较高水平HE4患者升高（中位生存期：17.3个月 *vs* 7.7个月，*P*＜0.05）。

国内学者也在此方面进行了探讨，于飞等^[17]^选取47例肺癌患者和31例健康体检者为研究对象，分析HE4在不同病理类型及TNM分期肺癌患者血清中的表达水平。结果显示，肺癌患者的血清HE4水平[（253.47±170.03）pmol/L]较健康体检者的血清HE4水平[（84.09±51.03）pmol/L]升高。不同病理类型及不同TNM分期的肺癌患者血清HE4水平差异均无统计学意义。血清HE4诊断肺癌的ROC曲线下面积（area under the ROC curve, AUC）为0.902，当HE4浓度为149.145 pmol/L时，其诊断肺癌的敏感度和特异度分别为72.3%和90.3%，检验效能高于CEA（AUC为0.765；当CEA浓度为4.685 μg/L时，敏感度和特异度分别为57.4%和83.9%）。因此认为，血清HE4对肺癌的诊断有较高的敏感度及特异度，检测患者血清HE4有助于诊断肺癌。

Yamashita等^[18]^通过免疫组化法对137例术后确诊的肺腺癌患者组织中HE4蛋白表达情况进行测定，发现41.6%的病例HE4呈阳性表达。在HE4表达阳性组中患者的5年无病生存率及5年总体生存率分别为44.6%和60.1%，对应的阴性组患者则分别达82.3%和90.8%，两组对比均有统计学差异（*P*=0.001）；在多变量分析时，HE4的阳性表达是患者无病生存期及总生存期较差的预测指标（HR=3.7, 95%CI: 1.7-8.4, *P*=0.001; HR=5.5, 95%CI: 1.8-17.2, *P*=0.003）。此外，研究表明，HE4的阳性表达与肿瘤组织病理分型无关，却与肺腺癌的亚型有关，腺癌患者HE4的阳性表达率（50%）明显高于支气管肺泡癌（22%）。在非支气管肺泡癌的腺癌患者中，HE4阳性组及阴性组患者5年无病生存率分别为41.6%和74.6%（*P*=0.03），5年整体生存率分别为54.1%和84.6%（*P*=0.02），经多变量分析，结节形态和HE4的表达为肺癌患者的独立预后指标。

## 3 HE4在恶性胸腔积液中的表达及意义

恶性胸腔积液是由胸内或胸外癌肿直接侵犯或转移至胸膜引起，肺癌是引起恶性胸腔积液最常见的病因，其他为乳腺癌、卵巢癌、胃癌、淋巴瘤等。Elsammak等^[19]^利用免疫测定技术对88例不同类型胸腔积液病人的胸水及血清HE4水平进行测定，其中漏出液患者22例，非肿瘤性渗出液患者32例，肿瘤性胸腔积液患者34例，其中23例由肺癌引起，2例结肠癌转移引起，2例非霍奇金淋巴瘤，2例胃癌转移，5例乳腺癌转移；23例肺癌患者中，11例为肺腺癌，12例鳞状细胞癌。实验结果表明，恶性组患者不管胸水还是血清中HE4水平均较漏出液组及非肿瘤性渗出液组升高。当胸水中HE4浓度诊断的临界值被定为1, 675 pmol/L可使恶性胸水的诊断敏感性达85.3%，特异性达90.7%，胸水HE4诊断恶性胸腔积液的AUC为0.886。此外，不同类型恶性肿瘤及不同病理分型的肺癌引起的恶性胸腔积液患者，血清及胸水中HE4含量比较无统计学差异（*P*＞0.05）。

HE4被发现后，引起广泛关注。它在多种肿瘤中高表达，并且成为卵巢癌的预测因子之一。但HE4在肺癌发生发展中的分子机制尚不清楚，年龄、孕期、吸烟、肾功能情况都可以影响血清HE4的检测，仍需有调整影响因素后的临床试验进一步验证其与肺癌发生发展的关系。而HE4在肺癌中其表达明显高于正常对照，特别是肺腺癌患者，其血清与胸腔积液中HE4增高。研究数据也显示其在诊断与随访中的重要作用。因此，HE4是潜在的肺癌诊断与预后因子。
